# Survey of Firearm Storage Practices and Preferences Among Parents and Caregivers of Children

**DOI:** 10.5811/westjem.21205

**Published:** 2024-11-27

**Authors:** Meredith B. Haag, Catlin H. Dennis, Steven McGaughey, Tess A. Gilbert, Susan DeFrancesco, Adrienne R. Gallardo, Benjamin D. Hoffman, Kathleen F. Carlson

**Affiliations:** * Doernbecher Children’s Hospital, Oregon Health and Science University, School of Medicine, and Doernbecher Injury Prevention Program Tom Sargent Safety Center, Department of Pediatrics, Portland, Oregon; † Oregon Health and Science University-Portland State University, School of Public Health, Portland, Oregon; ‡ Oregon Health and Science University, Department of Emergency Medicine, Portland, Oregon; ° Authors are co-primary authors

## Abstract

**Introduction:**

The American College of Emergency Physicians supports community- and hospital-based programs that intervene to prevent firearm-related injury. To this end, the distribution of firearm locks or storage devices in the emergency department (ED) may help achieve this target. To inform secure firearm storage programs for households with children and firearms, we examined firearm storage practices, device preferences, and cost tolerance among parents/caregivers of children.

**Methods:**

Between April 2018–November 2019, we conducted and analyzed an in-person survey of 294 caregivers, aged ≥18, with both children and firearms in the home. Surveys assessed reasons for firearm ownership, storage practices and device preferences among five storage-device options, and prices participants were willing to pay for devices. Practices and preferences were examined by participant characteristics. We used logistic regression to estimate odds ratios and 95% confidence intervals for associations of interest.

**Results:**

Most participants (73%) reported personal protection as a reason for owning firearms, and nearly 80% owned at least one firearm storage device. Over half (55%) owned cable locks, but only 36% of owners reported regularly using them. Rapid-access devices (electronic and biometric lockboxes) were less commonly owned (26%) but more likely to be regularly used (73%). The most highly rated storage device features were the following: the ability to store the firearm unloaded (87.3%); the ability to store the firearm loaded (79.1%); and device affordability (65%). Most participants (78%) preferred rapid-access devices over other options. Participants were willing to pay more for products that afforded rapid access to the firearm. Participants reported they would pay a median of $100 for a pushbutton rapid-access product ($80 retail), and $150 for a biometric lockbox ($210 retail).

**Conclusion:**

Understanding the storage practices and preferences among firearm-owning households with children can help inform ED injury-prevention screening and firearm safety practice implementation. Our results suggest that rapid-access devices may be the most preferable firearm storage devices for distribution by secure storage programs, and costs are likely minimal given parental/caregiver willingness to pay.

## INTRODUCTION

Firearm injuries are the single leading cause of death for children in the United States.^1^ Despite their inherent risk, firearms remain accessible to many children: estimates suggest that one in three US homes have at least one firearm, the majority of which are not stored securely.^2^ ^,^ ^3^ The American College of Emergency Physicians (ACEP) supports community- and hospital-based programs that intervene to prevent firearm-related injury. To this end, the distribution of firearm locks or storage devices in the emergency department (ED) may be effective.^4^ However, underutilization of these devices suggests that current distribution strategies do not meet the needs of firearm owners.^5^ ^–^ ^7^

Currently, there is limited knowledge about firearm storage preferences among caregivers of children. While prior literature has indicated that the costs of firearm safes can be a deterrent to their use, to our knowledge there is no published research regarding firearm owners’ willingness to pay for other types of storage devices or locks.^8^ ^,^ ^9^ This is critical to understand the costs and feasibility of implementation of firearm injury prevention programs. Our aim was to describe caregiver firearm owners’ preferences for, current use of, and willingness to pay for storage devices to inform prevention strategies.

## METHODS

### Study Design

We conducted an in-person, cross-sectional survey of firearm-owning parents/caregivers of children between April 2018–February 2019. We surveyed a convenience sample of caregivers of children at 10 community sites. These sites were geographically clustered in two major metropolitan areas with participants drawing mainly from two western states. Sites included an outdoor sporting activities fair, regional firearm show, academic children’s hospital safety center event, and multiple large community events across two metropolitan areas. Eligibility criteria for caregivers included the following: 1) aged ≥ 18 years; 2) having children (aged < 18 years) spending time in the home; 3) current or near-future possession of firearm(s); and 4) English language proficiency. Approval for the study was obtained from our local institutional review board (IRB#00017762).

### Procedures

Participants were recruited by tabling, and all surveys were conducted in person by members of the research team. Interested parents/caregivers, of whom 294 met eligibility criteria, completed an online consent form and anonymous survey electronically or on paper, which were stored via Research Electronic Data Capture (REDCap), hosted at Oregon Health & Science University.^10^ ^,^ ^11^

### Measures

Survey measures were adapted from an instrument used in a firearm storage preference survey at another institution.^7^ The survey assessed participants’ sociodemographic characteristics, firearm ownership, firearm and ammunition storage practices, and their storage device preferences and features that influenced their preference. The survey asked participants to consider five different firearm storage devices: 1) cable lock; 2) Life Jacket trigger lock; 3) lockbox with combination access; 4) electronic pushbutton-access lockbox; and 4) biometric-access (fingerprint) lockbox. Participants were asked to rate the importance of device features. The amount of money participants would be willing to pay for each device was then elicited using a sliding scale.

### Statistical Analyses

Using descriptive analyses, we examined participants’ storage practices, device preferences, and willingness to pay by their sociodemographic characteristics and reasons for firearm ownership. Medians are reported for the nonparametric willingness-to-pay data. Bivariable logistic regression analyses were performed to estimate associations between participant characteristics and likelihood of the following: 1) storing firearms locked; 2) storing firearms unloaded; and 3) storing ammunition locked separately at all times. Associations are reported as odds ratios (OR) with 95% confidence intervals (CI).

## RESULTS

### Study Sample

Participants predominantly were aged 25–34 (40.3%), White (81.1%) and owned both handguns and long guns (68.3%). Socioeconomic factors including income and education level were not statistically different among tabling sites (Table 1). Among those who responded to the open-ended question assessing primary reason for firearm ownership, most (72.7%) reported personal/home protection, while hunting/recreation was the exclusive reason for a minority (20.2%). We estimate a 15% response rate of all event participants and 40% of participants who were approached.^12^

**Table 1.** Characteristics of 259 survey participants, by most preferred firearm storage device.

		Preferred storage device					
	Total^a^ N (%)	Cable lock n (%)	Life jacket n (%)	Combination lockbox n (%)	Pushbutton lockbox n (%)	Biometric lockbox n (%)	*P* b
Total	259 (100)	13 (5.0)	7 (2.7)	12 (4.6)	48 (18.5)	152 (59.1)	-
Age	-	-	-	-	-	-	0.06
18–24	15 (6.3)	2 (13.3)	1 (6.7)	2 (13.3)	0	10 (66.7)	-
25–34	96 (40.3)	5 (5.4)	5 (5.4)	2 (2.2)	24 (26.1)	56 (60.9)	-
35–44	81 (34.0)	4 (5.2)	0	5 (6.5)	13 (16.9)	55 (71.4)	-
45+	46 (19.3)	1 (2.4)	1 (2.4)	2 (4.9)	10 (24.4)	27 (65.9)	-
Sex	-	-	-	-	-	-	**0.05**
Female	141 (56.9)	9 (6.9)	7 (5.3)	8 (6.1)	28 (21.4)	79 (60.3)	-
Male	107 (43.2)	3 (3.0)	0	4 (4.0)	20 (19.8)	74 (73.3)	-
Race	-	-	-	-	-	-	0.25
White	201 (81.1)	8 (4.3)	6 (3.2)	12 (6.4)	37 (19.7)	125 (66.5)	-
Other than White	47 (19.0)	4 (9.1)	1 (2.3)	0	11 (25.0)	28 (63.6)	-
Education	-	-	-	-	-	-	0.39
High school or less	34 (13.8)	3 (10.3)	1 (3.5)	4 (13.8)	2 (6.9)	19 (65.5)	-
Vocational school/some college	70 (28.3)	3 (4.5)	2 (3.0)	1 (1.5)	14 (20.9)	47 (70.2)	-
College	97 (39.3)	4 (4.4)	3 (3.3)	4 (4.4)	23 (25.0)	58 (63.0)	-
Graduate/professional school	46 (18.6)	2 (4.7)	1 (2.3)	3 (7.0)	9 (20.9)	28 (65.1)	-
Income	-	-	-	-	-	-	0.94
$49,999 or less	59 (22.8)	4 (7.4)	1 (1.9)	3 (5.6)	11 (20.4)	35 (64.8)	-
$50,000 or more	200 (77.2)	9 (5.0)	6 (3.4)	9 (5.0)	37 (20.7)	118 (65.9)	-
Military or law enforcement in home	-	-	-	-	-	-	0.62
Yes	53 (22.4)	3 (4.2)	1 (1.4)	3 (4.2)	19 (26.4)	46 (63.9)	-
No	184 (77.6)	8 (5.2)	6 (3.9)	9 (5.8)	28 (18.2)	103 (66.9)	-
Reason for firearm ownership	-	-	-	-	-	-	0.70
Personal protection only	83 (41.9)	3 (4.0)	3 (4.0)	3 (4.0)	18 (24.0)	48 (64.0)	-
Hunting/recreation only	40 (20.2)	3 (7.9)	1 (2.6)	2 (5.3)	7 (18.4)	25 (65.8)	-
Both	61 (30.8)	2 (3.4)	1 (1.7)	3 (5.1)	11 (18.6)	42 (71.2)	-
Other	14 (7.1)	2 (14.3)	1 (1.7)	1 (7.1)	4 (28.6)	6 (42.9)	-

a Total n may not equal sum of preferred storage device selections due to missing responses.

b All *P*-values based on Fisher exact test.

Boldface indicates statistical significance (*P* ≤ 0.05).

### Firearm Storage Practices

Nearly 80% of participants reported owning one or more of the five displayed firearm storage devices (Table 2). The most frequently owned device was a cable lock (55.3%); fewer owned rapid-access pushbutton or biometric storage devices (26.5% and 24.5%, respectively). Only 36.3% of cable lock owners reported always using their device, whereas 73.4% and 73.3% of pushbutton and biometric lockbox owners, respectively, reported always using their devices. Only 28.5% of participants reported compliance with American Academy of Pediatrics (AAP) recommendations, storing firearms unloaded and locked, and with ammunition locked separately. Those reporting firearm ownership for personal protection and those with a household member with current/past military/law enforcement service were less likely to practice secure storage (OR 0.4, 95% CI 0.2–0.7) and (OR 0.5, 95% CI 0.3–0.9), respectively (Table 1).

**Table 2.** Firearm storage device ownership, use, and willingness to pay thresholds.

	Cable lock	Life jacket	Combination lockbox	Pushbutton lockbox	Biometric lockbox
	n (%)	n (%)	n (%)	n (%)	n (%)
Own device	136 (55.3)	19 (7.6)	92 (37.1)	66 (26.5)	61 (24.5)
Use all the time^a^	49 (36.3)	6 (33.3)	49 (53.3)	47 (73.4)	44 (73.3)
Price willing to pay					
Median (IQR)	$20 (5–30)	$30 (15–50)	$50 (40–95)	$100 (60–120)	$150 (100–150)
% Retail cost	133%	67%	200%	125%	71%

a Among those who own respective device.

*IQR*, interquartile range.

### Storage Device Preferences

The largest proportions of participants endorsed biometric (59.1%) and pushbutton rapid-access (18.5%) lockboxes as their most preferred storage devices (Table 1). The most highly rated storage device features—with >60% of the sample indicating they were either “very important” or “absolutely essential”— were the ability to store the firearm unloaded (87.3%), the ability to store the firearm loaded (79.1%), and device affordability (65.0%).

### Willingness to Pay

Across demographic groups, participants were willing to pay more than retail price for combination lockboxes (median willingness to pay = $50, vs $25 actual retail cost) and pushbutton rapid-access lockboxes (median = $100, vs $80 retail; Table 2). For the most desired storage option, biometric lockboxes, survey participants indicated the highest willingness-to-pay dollar amount ($150 vs $210 retail).

## DISCUSSION

This study examines firearm storage-device preferences among firearm-owning parents/caregivers of children, describes how storage practices vary with their reasons for firearm ownership, and reports prices they are willing to pay for these devices. This unique data adds important context to interventions focused on the provision of firearm storage devices to families. Previous interventions have primarily focused on distributing cable locks, likely due to cost.^4^ ^,^ ^5^ ^,^ ^7^ However, there is limited data demonstrating sustained behavior change after distribution of cable locks, and surveys of firearm owners on device preference suggest they are undesirable.^6^ ^,^ ^9^ ^,^ ^13^ ^,^ ^14^ Results of our study suggest that most parents/caregivers who own firearms do so for reasons of personal protection, and that storage options that provide rapid access may be most desirable.

Our study continues to highlight the need for improved firearm storage in homes with children. Although 73.1% of respondents endorsed storing all firearms locked, adherence to other components of the AAP recommendations was low, with fewer than one-third of parents/caregivers in our study meeting all recommendations.

Firearm ownership among our participants was motivated by concerns about personal and family safety, consistent with prior studies.^15^ More than half of the sample (53.6%) identified the ability to store a firearm loaded as “absolutely essential.” Thus, it is not surprising that, while cable locks were the most frequently owned device, only one-third of cable lock owners reported regularly using them. In contrast, among rapid-access device owners, nearly three-fourths reported regular use. Rapid-access devices offer parents/caregivers an opportunity to securely store their firearms when not in use, which may lead to a higher prevalence of locked firearms and, potentially, reduced risk to children in these homes.

To prevent unintended access of firearms by children/youth in a home, preferred secure storage options must be financially attainable. While rapid-access devices are more expensive than other modalities, our respondents placed greater value on them, as demonstrated by the median amount they were willing to pay: $100 for push-button lockboxes and $150 for the biometric lockboxes. Since our survey collection, market forces have driven down costs, with biometric lockboxes routinely being sold for less than $100. This data has informed our institution’s own firearm storage device offerings by stocking biometric and rapid-access lockboxes. Future studies should focus on identifying groups that may benefit most from education on gun safety devices. This includes investigating the relationship between child age and motivation to use storage devices and expanding the survey into more geographically, racially, and socioeconomically diverse communities.

The ACEP states that “emergency physicians should advocate for evidence-based injury prevention policies.” Our data suggests that rapid-access storage devices are a desired and valued prevention strategy that can be employed by EDs. Integrated into a standard intake process, non-partisan discussion around firearm storage and providing access to these devices could be a meaningful method for EDs to meet advocacy goals.

## LIMITATIONS

There are several limitations of this research. First, this descriptive study involved a convenience sample of individuals attending healthcare appointments or community events in a predominantly urban area. However, we collected data from a wide variety of events to increase the diversity of participants. Second, our small sample size limited the precision of our estimates. Third, social desirability may have influenced participants’ responses. We mitigated this limitation by conducting anonymous surveys, which was emphasized during survey procedures. Lastly, our estimated response rates were low. This was mitigated by employing a short survey and offering a $5 gift card upon completion.

## CONCLUSION

Existing firearm storage-device distribution programs and practices may not meet the needs of all firearm owners. Parents/caregivers who own firearms may most prefer, and be most likely to use, rapid-access options. While these devices have higher costs, they are more likely to have sustained use and their costs are aligned with perceived value. We advocate that ED firearm intervention programs focus on devices that align with families’ priorities, which may improve sustained use and, ultimately, help decrease the rates of firearm-related mortality and morbidity among children.

## Supplementary Information





## References

[1] Centers for Disease Control and Prevention . Web-based injury statistics query and reporting system (WISQARS). 2024. Available from https://wisqars.cdc.gov/. Accessed May 1, 2024.

[2] Azrael D Cohen J Salhi C et al . Firearm storage in gun-owning households with children: results of a 2015 national survey. J Urban Health. 2018;95(3):295–304. 29748766 10.1007/s11524-018-0261-7 PMC5993703

[3] Johnson RM Coyne-Beasley T Runyan CW . Firearm ownership and storage practices, U.S. households, 1992–2002. A systematic review. Am J Prev Med. 2004;27(2):173–82. 15261906 10.1016/j.amepre.2004.04.015

[4] Rowhani-Rahbar A Simonetti JA Rivara FP . Effectiveness of interventions to promote safe firearm storage. Epidemiol Rev. 2016;38(1):111–24. 26769724 10.1093/epirev/mxv006

[5] Barkin SL Finch SA Ip EH et al . Is office-based counseling about media use, timeouts, and firearm storage effective? Results from a cluster-randomized, controlled trial. Pediatrics. 2008;122(1):e15–25. 18595960 10.1542/peds.2007-2611 PMC4486046

[6] Horn A Grossman DC Jones W et al . Community based program to improve firearm storage practices in rural Alaska. Inj Prev. 2003;9(3):231–4. 12966011 10.1136/ip.9.3.231 PMC1730982

[7] Simonetti JA Simeona C Gallagher C et al . Preferences for firearm locking devices and device features among participants in a firearm safety event. West J Emerg Med. 2019;20(4):552–6. 31316693 10.5811/westjem.2019.5.42727 PMC6625681

[8] Gaffley M Rauh JL Gardner A et al . Storage practices, devices, and presence of children among owners of firearms: informing pediatric firearm safety. Am Surg. 2023;89(12):5891–6. 37253678 10.1177/00031348231180932

[9] Anestis MD Moceri-Brooks J Johnson RL et al . Assessment of firearm storage practices in the US, 2022. JAMA Netw Open. 2023;6(3):e231447. 36862408 10.1001/jamanetworkopen.2023.1447 PMC9982690

[10] Harris PA Taylor R Minor BL et al . The REDCap consortium: building an international community of software platform partners. J Biomed Inform. 2019;95:103208. 31078660 10.1016/j.jbi.2019.103208 PMC7254481

[11] Harris PA Taylor R Thielke R et al . Research electronic data capture (REDCap)--a metadata-driven methodology and workflow process for providing translational research informatics support. J Biomed Inform. 2009;42(2):377–81. 18929686 10.1016/j.jbi.2008.08.010 PMC2700030

[12] Halpern S Asch D Shaked A et al . Standard definitions: final dispositions of case codes and outcome rates for surveys. Am J Transplant. 2005;5(6):1319–25. 15888036 10.1111/j.1600-6143.2005.00812.x

[13] KingA SimonettiJ BennettE et al . Firearm storage practices in households with children: a survey of community-based firearm safety event participants. Prev Med. 2020;131:105952.31843466 10.1016/j.ypmed.2019.105952

[14] Simonetti JA Simeona C Gallagher C et al . Preferences for firearm locking devices and device features among participants in a firearm safety event. West J Emerg Med, 2019;20(4):552–6. 31316693 10.5811/westjem.2019.5.42727 PMC6625681

[15] Cleveland EC Azrael D Simonetti JA et al . Firearm ownership among American veterans: findings from the 2015 National Firearm Survey. Inj Epidemiol. 2017;4(1):33. 29256160 10.1186/s40621-017-0130-y PMC5735043

[16] Buck-Atkinson J McCarthy M Stanley IH et al . Firearm locking device preferences among firearm owners in the USA: a systematic review. Inj Epidemiol. 2023;10(1):33. 37415242 10.1186/s40621-023-00436-7 PMC10326943

[17] National Academies of Sciences, Engineering, and Medicine . Integrating firearm injury prevention into health care: proceedings of a joint workshop of the National Academies of Sciences, Engineering, and Medicine; Northwell Health; and PEACE Initiative. Washington, DC: The National Academies Press; 2022. 36626591

