# Supplementary material for: Survey of Firearm Storage Practices and Preferences Among Parents and Caregivers of Children
Source: West J Emerg Med. 2024 Nov 27;26(1):142–6. doi: 10.5811/westjem.21205 (PMC11908524; doi:10.5811/westjem.21205)
Supplement: Supplementary file 1 [file wjem-26-142-s001.pdf]

Anonymous Safety Device Preference Survey

Where was this survey completed?

◯ DCH lobby
◯ Baby Fest
◯ Community Event
◯ Other

Notes about location:

Today's Date

____

Click "next page" to proceed to the survey.

Preference Survey

This brief anonymous survey was developed to better understand what types of gun storage devices people in the Portland-metro community would use to help keep themselves and their families safe.

Your responses to this survey will be used in aggregate which means your responses to this survey will be combined with other individuals' responses to see trends in preference. In other words, your answers will not be reported individually.

Thank you so much for taking the time to answer these questions! Your responses will help us understand what the community would like, and what they need, in an effort to keep children and families safe.

Are you a parent?

◯ Yes
◯ No

Do children live in or spend time in your home?

◯ Yes
◯ No

Will children live in or spend time in your home in the near future?

◯ Yes
◯ Maybe
◯ No

Are there guns present in your home now?

◯ Yes
◯ No

Will guns be present in your home in the near future?

◯ Yes
◯ Maybe
◯ No

Gun Safe Preference Questions

There are many options for safely storing guns. On the following pages are a few examples of these options. We are interested in learning about your thoughts and attitudes about each of them.

We will be asking you to tell us how much you would be willing to pay for each device. You will be shown a text box that you can enter any number within a given range. Please enter only a whole number (ex: 1 vs. 1.25) without a dollar sign.

Device A: Cable Lock

Device A: Cable Lock

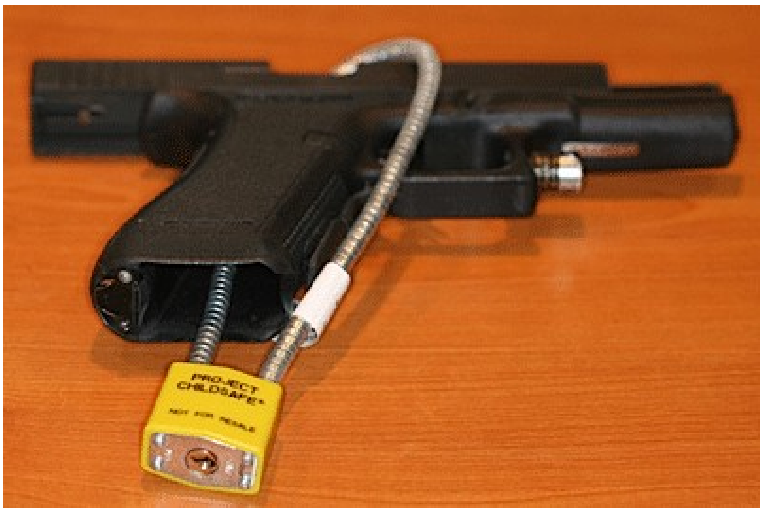

Device A: Cable Lock

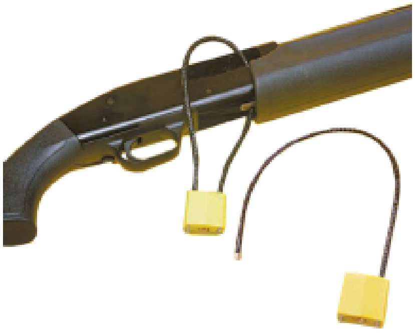

How much would you be willing to pay for this device?
Your response must be between $0 and $150. Enter just the dollar amount you would be willing to pay, do not include a dollar sign.

____

Device A: Cable Lock

Device A: Cable Lock

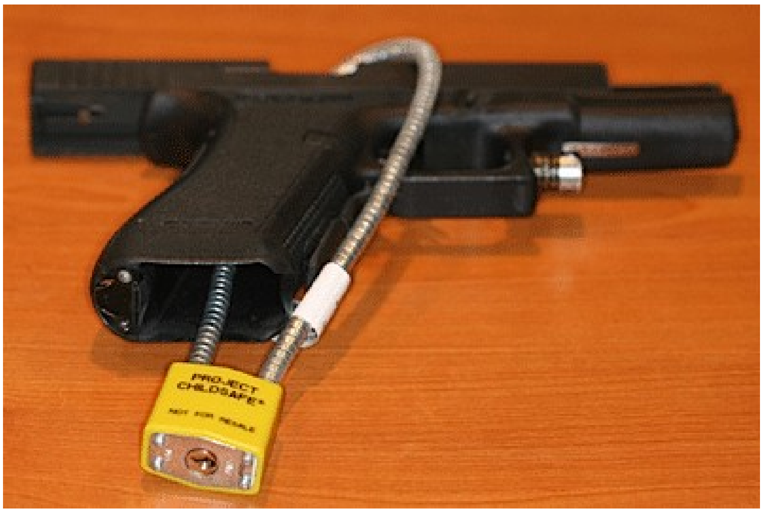

Device A: Cable Lock

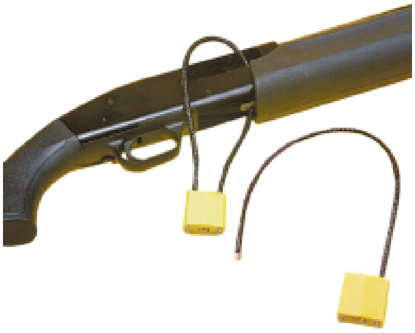

The retail cost (how much it would cost in a store) of this device is around $10 or less. How much would you be willing to pay for this device?
Your response must be between $0 and $10. Enter just the dollar amount you would be willing to pay, do not include a dollar sign.

____

Do you currently own this device (or a similar device)?

◯ Yes
◯ No

Do you currently use this device for storing your gun?

◯ No
◯ Yes, all of the time
◯ Yes, most of the time
◯ Yes, some of the time

Why do you not use this device?
(Select all that apply.)

☐ The device is difficult to use
☐ The device is broken
☐ I forget to use this device
☐ I use another device instead
☐ The device prevents me from quickly accessing my gun
☐ I can't find the device
☐ Other

Please select the answer that best describes how you feel about this device:

◯ I would NEVER USE this device if I owned one
◯ I would CONSIDER USING this device if I owned one
◯ I would DEFINITELY USE this device if I owned one

Device B: "Life Jacket" Locking Device

Device B: "Life Jacket" Locking Device

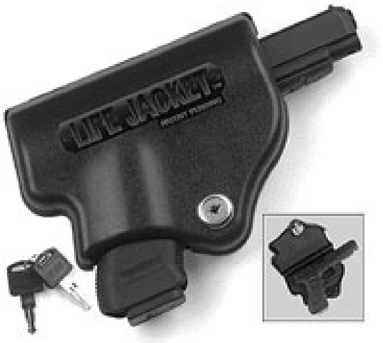

Device B: "Life Jacket" Locking Device

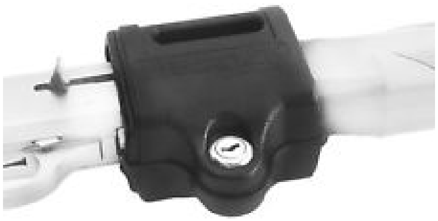

How much would you be willing to pay for this device?
Your response must be between $0 and $150. Enter just the dollar amount you would be willing to pay, do not include a dollar sign.

____

Device B: "Life Jacket" Locking Device

Device B: "Life Jacket" Locking Device

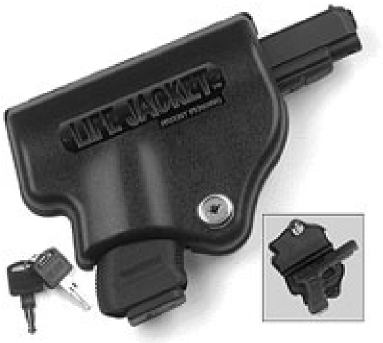

Device B: "Life Jacket" Locking Device

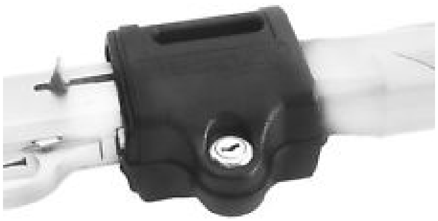

The retail cost (how much it would cost in a store) of this device is around $20 - $30. How much would you be willing to pay for this device?
Your response must be between $0 and $30. Enter just the dollar amount you would be willing to pay, do not include a dollar sign.

____

Do you currently own this device (or a similar device)?

○ Yes
○ No

Do you currently use this device for storing your gun?

○ No
○ Yes, all of the time
○ Yes, most of the time
○ Yes, some of the time

Why do you not use this device?
(Select all that apply.)

☐ The device is difficult to use
☐ The device is broken
☐ I forget to use this device
☐ I use another device instead
☐ The device prevents me from quickly accessing my gun
☐ I can't find the device
☐ Other

Please select the answer that best describes how you feel about this device:

◯ I would NEVER USE this device if I owned one
◯ I would CONSIDER USING this device if I owned one
◯ I would DEFINITELY USE this device if I owned one

Device C: Lock Box with Keyed Access (requires a key to open it)

Device C: Lock Box with Keyed Access (requires a key to open it)

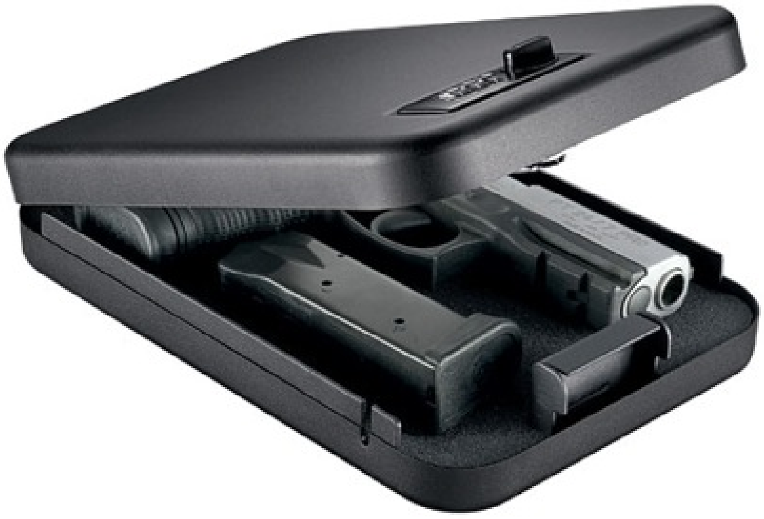

How much would you be willing to pay for this device?
Your response must be between $0 and $150. Enter just the dollar amount you would be willing to pay, do not include a dollar sign.

____

Device C: Lock Box with Keyed Access (requires a key to open it)

Device C: Lock Box with Keyed Access (requires a key to open it)

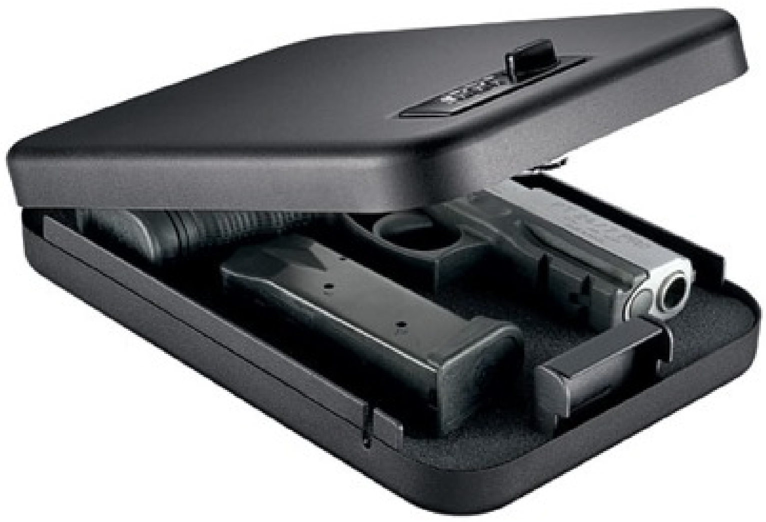

The retail cost (how much it would cost in a store) of this device can range from $20 to $100, depending on the features it comes with. How much would you be willing to pay for an average version of this device, as pictured in the image above?
Your response must be between $0 and $100. Enter just the dollar amount you would be willing to pay, do not include a dollar sign.

____

Do you currently own this device (or a similar device)?

◯ Yes
◯ No

Do you currently use this device for storing your gun?

◯ No
◯ Yes, all of the time
◯ Yes, most of the time
◯ Yes, some of the time

Why do you not use this device?
(Select all that apply.)

☐ The device is difficult to use
☐ The device is broken
☐ I forget to use this device
☐ I use another device instead
☐ The device prevents me from quickly accessing my gun
☐ I can't find the device
☐ Other

Please select the answer that best describes how you feel about this device:

◯ I would NEVER USE this device if I owned one
◯ I would CONSIDER USING this device if I owned one
◯ I would DEFINITELY USE this device if I owned one

Device D: Quick Access Electronic Gun Safe (uses a key pad to open, with a key for back up)

Device D: Quick Access Electronic Gun Safe (uses a key pad to open, with a key for back up)

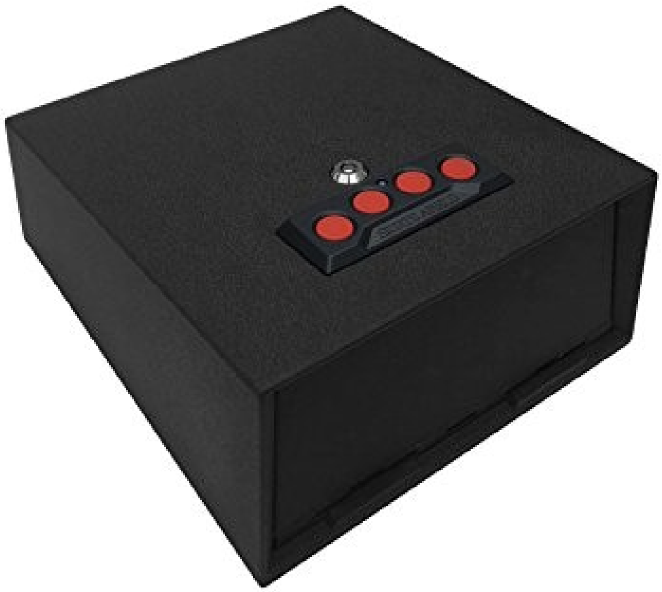

How much would you be willing to pay for this device?
Your response must be between $0 and $150. Enter just the dollar amount you would be willing to pay, do not include a dollar sign.

____

Device D: Quick Access Electronic Gun Safe (uses a key pad to open, with a key for back up)

Device D: Quick Access Electronic Gun Safe (uses a key pad to open, with a key for back up)

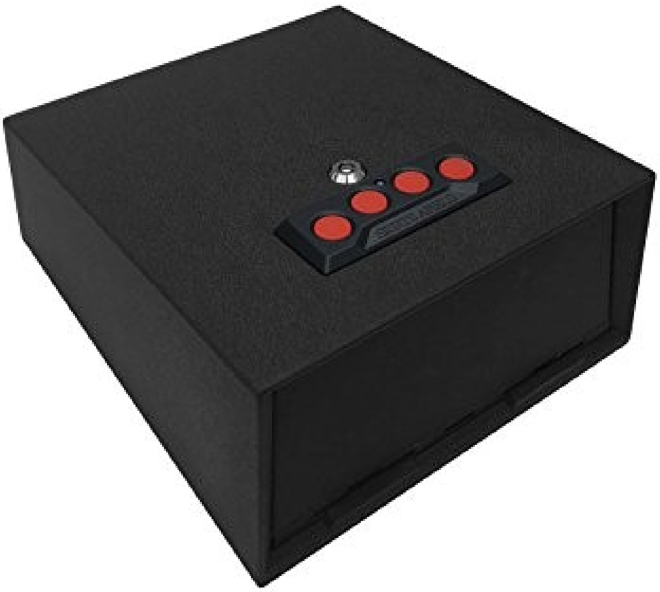

The retail cost (how much it would cost in a store) of this device can range from $100 to $200, depending on the features it comes with. How much would you be willing to pay for an average version of this device, as pictured in the image above?
Your response must be between $0 and $200. Enter just the dollar amount you would be willing to pay, do not include a dollar sign.

____

Do you currently own this device (or a similar device)?

◯ Yes
◯ No

Do you currently use this device for storing your gun?

◯ No
◯ Yes, all of the time
◯ Yes, most of the time
◯ Yes, some of the time

Why do you not use this device?
(Select all that apply.)

☐ The device is difficult to use
☐ The device is broken
☐ I forget to use this device
☐ I use another device instead
☐ The device prevents me from quickly accessing my gun
☐ I can't find the device
☐ Other

Please select the answer that best describes how you feel about this device:

◯ I would NEVER USE this device if I owned one
◯ I would CONSIDER USING this device if I owned one
◯ I would DEFINITELY USE this device if I owned one

Device E: Gun Safe - Biometric (Fingerprint technology)

Device E: Gun Safe - Biometric (Fingerprint technology)

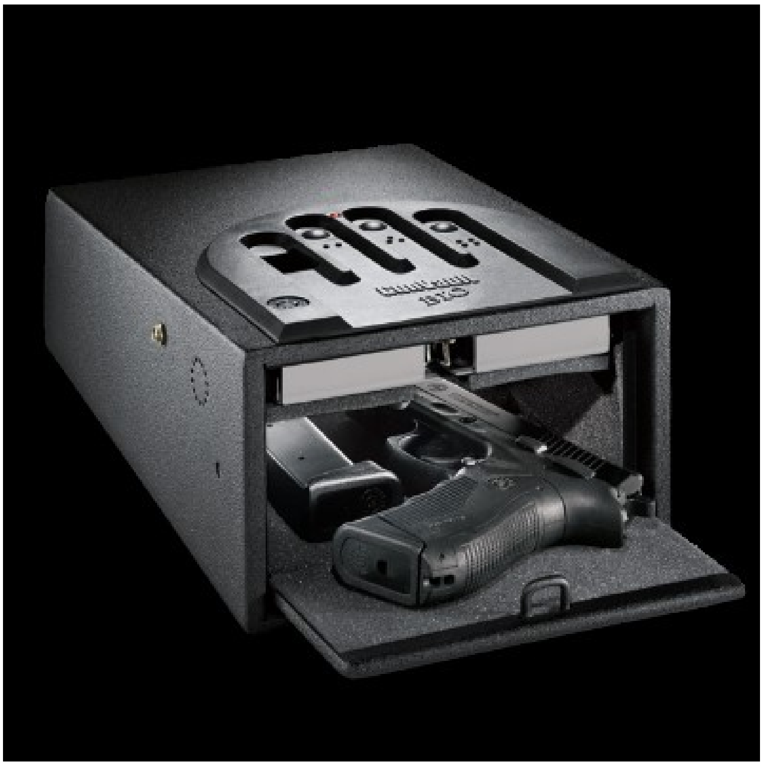

Device E: Gun Safe - Biometric (Fingerprint technology)

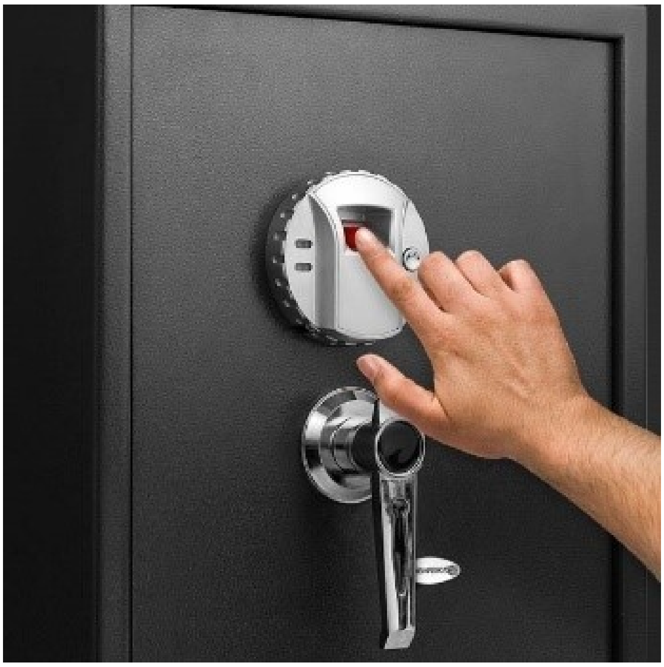

How much would you be willing to pay for this device?
Your response must be between $0 and $150. Enter just the dollar amount you would be willing to pay, do not include a dollar sign.

____

Device E: Gun Safe - Biometric (Fingerprint technology)

Device E: Gun Safe - Biometric (Fingerprint technology)

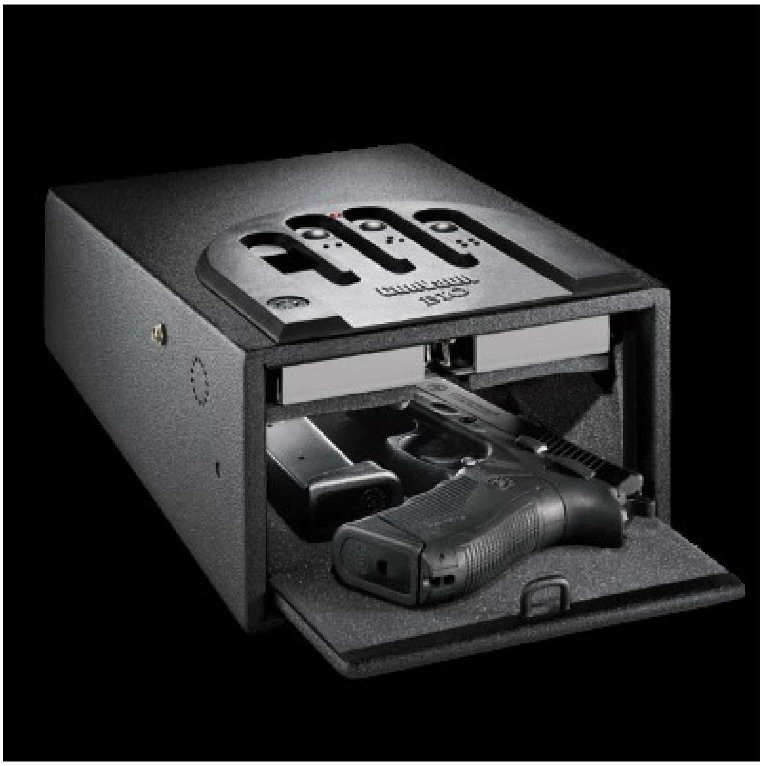

Device E: Gun Safe - Biometric (Fingerprint technology)

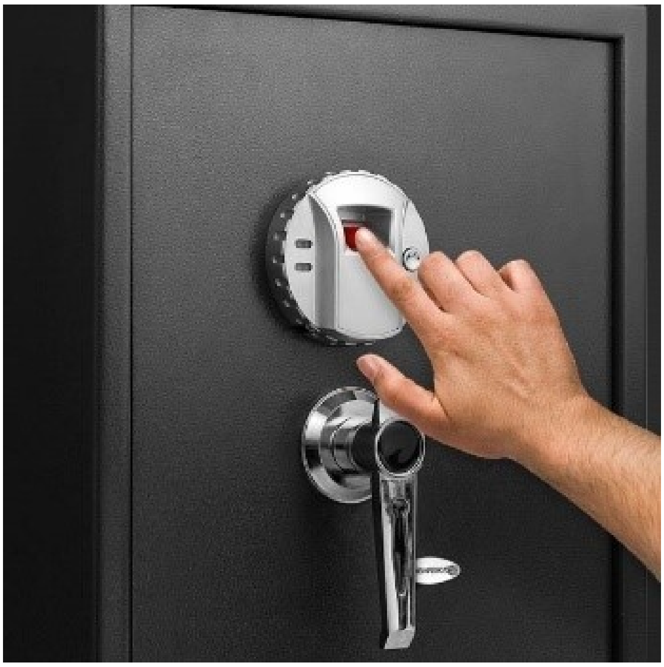

The retail cost (how much it would cost in a store) of this device can range from $80 to over $400, depending on the features it comes with. How much would you be willing to pay for an average version of this device, as pictured in the image above?
Your response must be between $0 and $400. Enter just the dollar amount you would be willing to pay, do not include a dollar sign.

____

Do you currently own this device (or a similar device)?

◯ Yes
◯ No

Do you currently use this device for storing your gun?

◯ No
◯ Yes, all of the time
◯ Yes, most of the time
◯ Yes, some of the time

Why do you not use this device?
(Select all that apply.)

☐ The device is difficult to use
☐ The device is broken
☐ I forget to use this device
☐ I use another device instead
☐ The device prevents me from quickly accessing my gun
☐ I can't find the device
☐ Other

Please select the answer that best describes how you feel about this device:

◯ I would NEVER USE this device if I owned one
◯ I would CONSIDER USING this device if I owned one
◯ I would DEFINITELY USE this device if I owned one

Device Feature Preferences

Please tell us how important each of these features is to you for a gun storage device:

If you don't currently own a gun, please imagine that you are a gun owner, and think about what features would be important to you in a gun storage device.

	Not Important at All	Of Little Importance	Of Average Importance	Very Important	Absolutely Essential
The ability to quickly lock and unlock the gun	○	○	○	○	○
The device is affordable	○	○	○	○	○
The ability to lock the gun when it is loaded	○	○	○	○	○
The color and overall appearance of the device	○	○	○	○	○
The device can be unlocked using a key	○	○	○	○	○

Device Feature Preferences (continued)

Please tell us how important each of these features is to you for a gun storage device:

If you don't currently own a gun, please imagine that you are a gun owner, and think about what features would be important to you in a gun storage device.

	Not Important at All	Of Little Importance	Of Average Importance	Very Important	Absolutely Essential
The device can be unlocked with a number combination	○	○	○	○	○
The device can be unlocked using fingerprint technology	○	○	○	○	○
The device can be easily moved between your household and vehicle	○	○	○	○	○
The device can be used on both handguns and long guns (like a rifle)	○	○	○	○	○
The device has been recommended for use by police or gun advocacy organizations	○	○	○	○	○

Device Ranking by Preference

If money wasn't an issue, please rank the following safety devices from your most favored choice (1st choice) to your least favored choice (5th choice).

**Device A:** Cable Lock	**Device B:** "Life Jacket" Locking Device	**Device C:** Lock Box with Keyed Access (requires a key to open)
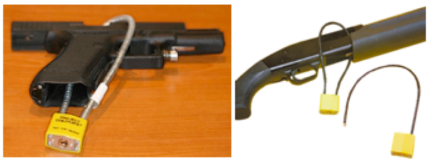	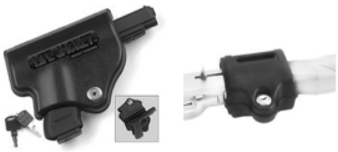	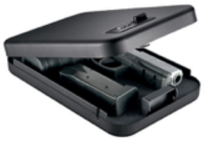
**Device D:** Quick Access Electronic Gun Safe (uses a key pad to open with a key for back up)	**Device E:** Gun Safe – Biometric (fingerprint technology)	
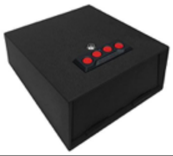	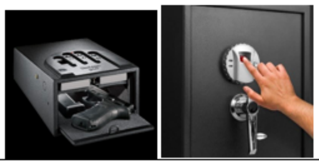	

	1st choice (most favored)	2nd choice	3rd choice	4th choice	5th choice (least favored)
Device A: Cable lock	○	○	○	○	○
Device B: Life Jacket lock	○	○	○	○	○
Device C: Gun lockbox with key access	○	○	○	○	○
Device D: Gun lockbox with electronic keypad access	○	○	○	○	○
Device E: Gun safe/lockbox with biometric (fingerprint) access	○	○	○	○	○

About You

These first questions will help us better understand who is taking the survey. They will not be used to try to identity you as a participant.

Who lives in your household? Please select all that apply.

☐ Spouse
☐ Significant other
☐ Children (ages 18 or younger)
☐ Children (over the age of 18)
☐ Family members outside of my immediate family (e.g. grandparents, cousins, etc.)
☐ Non-family members (e.g. renters, friend, etc.)
☐ I live alone

How many children live in your home or spend time in your home?

◯ 1
◯ 2
◯ 3
◯ 4
◯ 5
◯ More than 5

How often do you have children in your home? If your child's only residence is your home, please select "Daily."

(These don't need to be your children, this could apply to neighbors, nieces/nephews, etc.).

◯ Never
◯ Rarely (less than once a month)
◯ Monthly
◯ Weekly
◯ Daily

What are the ages of your children? Please select all that apply.

(For example: If you have an infant, a 2 year old, and a 9 year old, you would select a, b, and d)

☐ a) Under a year
☐ b) 1-4 years
☐ c) 5-7 years
☐ d) 8-10 years
☐ e) 11-13 years
☐ f) 14-18 years

What is your age? Please select the age bracket you fit in.

◯ 18-24 years old
◯ 25-34 years old
◯ 35-44 years old
◯ 45-54 years old
◯ 55-64 years old
◯ 65-74 years old
◯ 75 years or older

What is your gender?

◯ Female
◯ Male
◯ Non-binary

How would you describe your race/ethnicity? Please select all that apply.

- ☐ White or Caucasian
- ☐ Black or African American
- ☐ Hispanic or Latino/a
- ☐ Asian
- ☐ American Indian or Alaskan Native
- ☐ Native Hawaiian or Pacific Islander
- ☐ Middle Eastern or Arab
- ☐ Other

What is the primary language spoken in your home?

- ◯ English
- ◯ Spanish
- ◯ Vietnamese
- ◯ Chinese
- ◯ Russian
- ◯ Arabic
- ◯ Other

What is the highest grade of school you have completed?

- ◯ High school or less
- ◯ Vocational school/some college
- ◯ College
- ◯ Graduate/Professional school

Have you ever served in the US Armed forces?

- ◯ Yes
- ◯ No

Has anyone in your home ever served in the US Armed Forces?

- ◯ Yes
- ◯ No

Are you or someone in your home a law enforcement or public safety officer?

- ◯ Yes
- ◯ No

What type of insurance does your child (or children) have?

- ◯ Medicaid
- ◯ CHIP
- ◯ Private insurance (for example: Aetna, Blue Cross, United, Kaiser, Moda, etc).
- ◯ Uninsured
- ◯ Don't know
- ◯ Not applicable, I don't have a child

What is your household's total annual income?

◯ $25,000 or less
◯ $25,001 - $49,999
◯ $50,000 - $79,999
◯ $80,000 - $119,999
◯ $120,000 - $149,000
◯ $150,000 or more

Questions Regarding Gun Ownership

This next set of questions will help give us a better understanding of what types of guns are present in most homes in this area.

What would you say is the primary reason why a gun or guns are present in your home?

What types of guns do you own?
(Select all that apply.)

☐ Handgun/s (pistol, revolver)
☐ Long gun/s (rifle, shotgun)
☐ Other

How would you describe this other gun/s?

Are the guns in your home currently stored locked?
Examples include using a locked cabinet, locked box, trigger lock or cable gun lock.

◯ Yes, all of them
◯ Yes, some of them
◯ None of them are locked
◯ Not sure

What percentage of the time would you say your gun or guns are locked?

None of the time | Half of the time | All of the time

(Place a mark on the scale above)

What types of locks do you currently use? Please select all that apply.

☐ Locked cabinet, closet, etc.
☐ Lock box or safe
☐ Trigger lock
☐ Cable gun lock
☐ Lifejacket lock
☐ Other

How would you describe this other storage?

Are the guns in your home currently stored loaded?

◯ Yes, all of them
◯ Yes, some of them
◯ None of them are loaded
◯ Not sure

What percentage of the time would you say your gun or guns are stored loaded?

None of the time   Half of the time   All the time

(Place a mark on the scale above)

Is the ammunition in your home currently stored separately in a locked place?

◯ No ammunition is in my home
◯ Yes, all of it
◯ Yes, some of it
◯ None of the ammunition is locked
◯ Not sure

What percentage of the time would you say your ammunition is locked separately from the gun?

None of the time   Half of the time   All the time

(Place a mark on the scale above)
